# Sand-Fly Saliva-*Leishmania*-Man: The Trigger Trio

**DOI:** 10.3389/fimmu.2013.00375

**Published:** 2013-11-19

**Authors:** Fabiano Oliveira, Augusto M. de Carvalho, Camila I. de Oliveira

**Affiliations:** ^1^Vector Biology Section, Laboratory of Malaria and Vector Research, National Institute of Allergy and Infectious Diseases, National Institutes of Health, Rockville, MD, USA; ^2^Laboratório de Imunoparasitologia, Centro de Pesquisas Gonçalo Moniz, FIOCRUZ, Salvador, Brazil; ^3^Instituto Nacional de Ciência e Tecnologia de Investigação em Imunologia (iii-INCT), Salvador, Brazil

**Keywords:** sand-fly, saliva, *Leishmania*, vaccine, leishmaniasis

## Abstract

Leishmaniases are worldwide diseases transmitted to the vertebrate host by the bite of an infected sand-fly. Sand-fly biting and parasite inoculation are accompanied by the injection of salivary molecules, whose immunomodulatory properties are actively being studied. This mini review focuses on how the interactions between sand-fly saliva and the immune system may shape the outcome of infection, given its immunomodulatory properties, in experimental models and in the endemic area. Additionally, we approach the recent contributions regarding the identification of individual salivary components and how these are currently being considered as additional components of a vaccine against leishmaniasis.

## Background

Leishmaniases are a widespread group of diseases caused by *Leishmania* protozoa. Clinical manifestations range from skin ulcers to fatal visceral disease (Table 1). *Leishmania* parasites are injected into the vertebrate host upon sand-fly biting and take up permanent residence within macrophages, where they replicate and cause disease. At the moment of parasite inoculation, the vertebrate host is simultaneously injected with sand-fly saliva, which causes vasodilation, prevents blood clotting (1), and host hemostasis. This facilitates blood feeding, ultimately needed for egg maturation; however, salivary molecules also modulate the vertebrate host’s immune response. This mini review describes the immunomodulatory properties of sand-fly saliva and how they play on *Leishmania* infection, contributing to the outcome of infection and, in parallel, suggesting intervention strategies such as vaccination. Moreover, high-throughput methodologies have enabled a precise description of salivary gland transcriptomes or “sialomes.” We also describe some of these molecules and their distribution within different sand-fly species.

**Table 1:** **Clinical forms, main reservoirs, and sand-fly vectors of various *Leishmania* species**.

*Leishmania* species	Usual clinical forms	Main reservoirs	Vector sand-fly species
*Leishmania infantum chagasi*	VL	Dogs	*Lutzomyia longipalpis, Lutzomyia evansi*
		Humans	
*Leishmania infantum*	VL, CL	Dogs	*Papatasi pernicious, Papatasi ariasi, Papatasi tobbi, Papatasi neglectus*
*Leishmania donovani*	VL	Humans	*Papatasi argentipes, Papatasi orientalis, Papatasi martini*
*Leishmania tropica*	CL	Humans	*Papatasi sergenti*
*Leishmania major*	CL	Rodents	*Papatasi papatasi, Papatasi duboscqi, Papatasi salehi, Papatasi bergeroti*
*Leishmania braziliensis*	CL, ML	Dogs	*Lutzomyia intermedia, Lutzomyia whitmani, Lutzomyia migonei, Lutzomyia wellcomei, Lutzomyia ovallesi*
		Humans	
		Rodents	
*Leishmania amazonensis*	CL, DCL	Rodents	*Lutzomyia flaviscutellata*
*Leishmania guyanensis*	CL, ML	Marsupials	*Lutzomyia umbratilis, Lutzomyia anduzei, Lutzomyia whitmani*
		Rodents	
*Leishmania mexicana*	CL, DCL	Rodents	*Lutzomyia olmeca olmeca, Lutzomyia shannoni, Lutzomyia diabolica*
		Marsupials	

*VL, visceral leishmaniasis; CL, cutaneous leishmaniasis; ML, mucosal leishmaniasis; DCL, diffuse cutaneous leishmaniasis*.

## Salivary Components Modulate the Immune System

In the late 1980s, early 90s, studies showed that co-inoculation of *Lutzomyia longipalpis* sand-fly salivary gland sonicate (SGS), employed as surrogate of sand-fly salivary components, enhanced experimental infection by *Leishmania* sp. parasites (2, 3). SGS from *Phlebotomus duboscqi*, a vector for *Leishmania major*, was chemoattractive for mouse monocytes (4) whereas SGS from *Phlebotomus papatasi*, another vector of *L. major*, inhibited macrophage activation by IFN-γ (5) and downregulated Inducible Nitric Oxide Synthase (iNOS) expression, thereby reducing NO production in murine macrophages (6). These results imply that *Leishmania* parasites exploit these effects to ascertain survival within infected host cells.

Belkaid et al. (7) developed a “natural model” of infection in which co-inoculation of mice with *L. major* parasites plus *P. papatasi* SGS converted C57BL/6 mice – naturally resistant to *L. major* infection – into a non-healing phenotype. This outcome was associated with an increase in Th2-related cytokines such as IL-4 and IL-5. CBA mice co-inoculated with *L. major* parasites and *P. papatasi* SGS also upregulated expression of IL-4 and reduced production of IFN-γ, IL-12, and iNOS (8), promoting parasite proliferation. Following these observations, a series of studies further explored mechanisms involving enhanced *Leishmania* infection in the presence of sand-fly SGS: upon co-inoculation of *L. amazonensis* plus *L. longipalpis* SGS, larger lesions developed and were associated with elevated IL-10 production by draining lymph node cells stimulated *in vitro* with SGS (9). IL-10 suppresses effector functions of monocytes and macrophages and NO and H_2_O_2_ production (10), molecules that promote *Leishmania* killing. Moreover, *L. longipalpis* SGS recruited macrophages *in vitro*, promoting parasite survival and proliferation (11), an effect dependent on production of CCL2 (12). This observation was later confirmed *in vivo* following exposure of mice to *L. longipalpis* bites (13) or stimulation of the peritoneal cavity with *L. major* plus *L. longipalpis* SGS (14), which also resulted in IL-10 production.

In a mouse model of OVA-induced peritonitis, pre-treatment with *L. longipalpis* SGS reduced neutrophil recruitment by inhibiting production of TNF-α and IL-1b (15). *P. papatasi* or *P. duboscqi* SGS also reduced MHC Class II expression by dendritic cells and induced IL-10 and Prostaglandin E_2_ (PGE_2_) production (16). Intraperitoneal injection of *L. longipalpis* SGS again increased the production of PGE_2_ and Leukotriene B_4_ (LTB_4_) and, in parallel, triggered the formation of lipid bodies (17). Indeed, PGE_2_ has been implicated in macrophage infection with *Leishmania* (18), suggesting that lipid mediators may be another parasite strategy to escape killing and establish infection. Of note, salivary molecules also modulate the function of neutrophils: *L. longipalpis* SGS triggers neutrophil apoptosis shown by caspase activation and expression of FasL (19) and were associated with increased parasite survival, an effect counteracted by a caspase inhibitor.

Maxadilan, a potent vasodilator, was the first molecule identified in sand-fly saliva (20, 21). It inhibits proliferation of mouse lymphocytes *in vitro* (22) and decreases TNF-α production both *in vivo* (23) and in lipopolysaccharide-treated mouse macrophages (24). Maxadilan alone exacerbated *L. major* infection to the same degree as whole SGS (25) due to its capacity to upregulate the production of IL-10 and TGF-β and, in parallel, suppress IL-12p40, TNF-α, and NO production (26). Employing a model of Collagen-Induced Arthritis (CIA), Carregaro et al. (27), showed that *P. papatasi* SGS administered daily attenuated disease severity, an effect associated with adenosine and 5′ AMP (28), both of which are present at pharmacologic levels within sand-fly saliva. LJM11, a protein present in *L. longipalpis* SGS, prevented neutrophil migration triggered by antigen challenge in OVA-immunized mice (29). In parallel, TNF-α expression was reduced and IL-10 secretion was increased. Collectively, these results highlight the possibility of exploring salivary molecules in therapy against inflammatory diseases. Indeed, both Maxadilan and *P. papatasi* SGS decreased IFN-γ and IL-12p40 production by human Peripheral Blood Mononuclear Cells (PBMCs) and increased IL-6 secretion *in vitro* (30). Treatment of lipopolysaccharide-stimulated human monocytes with *L. longipalpis* saliva confirmed its ability to decrease TNF-α production whereas differentiation of monocyte-derived dendritic cells in the presence of SGS inhibited expression of co-stimulatory molecules (31).

## Salivary Molecules as Vaccine Candidates?

Following the observation that mice pre-exposed to *P. papatasi* SGS were protected against a challenge infection (7), a possibility emerged that a raised immune response to saliva could counteract its “exacerbative” properties and, hence, confer protection against disease. Indeed, mice repeatedly exposed to uninfected sand flies mounted an anti-saliva immune response that prevented lesion development (32). Authors associated this outcome with the development of a Delayed-Type Hypersensitivity (DTH) reaction and with IFN-γ production, the latter acting as the hallmark cytokine associated with protection against *Leishmania*. Soon after, PpSP15 was identified in *P. papatasi* saliva, and mice immunized with the corresponding DNA plasmid were protected against a challenge infection with *L. major* (33). Importantly, B cell-deficient mice were also protected, suggesting that antibodies were not required for protection.

Similarly, immunization with *L. longipalpis* LJM19 protected against infection by *Leishmania infantum chagasi*, in a model of Visceral Leishmaniasis (VL) (34). Again, protection correlated with a high IFN-γ:TGF-β ratio and with elevated iNOS expression in the liver of challenged hamsters. In dogs, the main reservoirs of VL in Latin America, immunization with two other proteins found in *L. longipalpis* saliva – namely LJM17 or LJM143 – followed by exposure to sand-fly bites led to enhanced IL-12 and IFN-γ production (35). Additionally, lymphocytes from immunized dogs effectively killed parasites *in vitro*. In a recent study, mice immunized with recombinant LJM11, present in *L. longipalpis* saliva, in the absence of an adjuvant, were challenged by *L. major*-infected sand-fly bites (36). Authors detected IFN-γ being produced in response to LJM11 vaccination, which correlated with decreased numbers of parasites. Collectively, these studies strengthen the concept that anti-saliva immunity can be exploited in the context of *Leishmania* vaccine development.

In fact, the combination of a salivary molecule with a *Leishmania* antigen for vaccine development has been pursued in different experimental models. Immunization of hamsters with DNA plasmids encoding LJM19, present in *L. longipalpis* saliva, plus KMP-11 [a *Leishmania*-derived candidate molecule for vaccine development (37)] reduced the parasite load after a challenge with *L. infantum chagasi* (38). This effect was similar to that observed upon immunization with LJM19 or with KMP-11 alone. Immunization of hamsters with the same DNA plasmid coding for LJM19 also prevented disease development following challenge with *Leishmania braziliensis* plus *Lutzomyia intermedia* (a vector for *L. braziliensis*) (see Table 1) SGS, expanding the possibility of using single salivary molecules to induce immunity to disease caused by different species of *Leishmania* (39). Actually, immunization with *L. longipalpis* SGS resulted in an expansion of CD4^+^ and CD8^+^ T cells that secrete IFN-γ and lead to reduced *L. braziliensis* infection in mice (40). Collectively, these studies point that immunization with certain sand-fly salivary components induces the Th1-biased immune response needed to control infection by *Leishmania*, currently reviewed elsewhere (41).

One major criticism to the works cited above is that animals were challenged with SGS and *Leishmania* by needle inoculation and were not submitted to the stringent conditions of natural transmission of *Leishmania*, that is, the sand-fly bite. Real-time microscopy showed that sand-fly biting at a dermal site in the mouse recruits a massive influx of neutrophils (42). Additionally, *L. major* parasites remained viable following phagocytosis by invading neutrophils, hinting at an immune evasion strategy to ascertain infection. Therefore, there is a pressing need to test vaccine candidates in a “real life” scenario, i.e., challenge immunized mice by bites of infected sand flies. This was the context in which Gomes et al. evaluated whether vaccination with KSAC (43) or L110f (44) – two candidates for a leishmaniasis vaccine – conferred protection against *L. major* transmission by sand-fly bites (45). Following sand-fly challenge, only KSAC plus GLA-SE (a synthetic TLR-4 agonist employed as adjuvant)-immunized mice showed a significant reduction of parasite number, whereas parasite levels in L110f-immunized mice were not significantly different from controls. Protection in KSAC-immunized mice correlated with IFN-γ-secreting CD4^+^ T cells as seen in another study in which mice were immunized with recombinant *L. donovani* superoxide dismutase B1 plus GLA-SE (46). These results suggest that vaccine candidates that perform well against live *Leishmania* infection may be further investigated in experiments involving challenge by sand-fly biting.

Moreover, following exposure to infected sand flies, mice that spontaneously healed a primary infection by *L. major* displayed a significantly lower parasite load and higher percentage of IFN-γ-secreting CD4^+^ T cells compared with mice immunized with KSAC plus adjuvant (47). Indeed, healed mice are resistant to parasite transmission by sand flies (48), and this was associated with rapid mobilization of CD4^+^ T cells specific to *Leishmania* to the challenge site, hampering establishment of disease. Although these observations highlight the need to pursue studies with infected sand flies, it must be emphasized that this possibility is currently restricted to few laboratories in the world.

## The Distinguished Case of *L. intermedia*, a Vector for *L. braziliensis* in Brazil

Early studies showed that *L. braziliensis* infection was enhanced in the presence of sand-fly saliva (49–52). From these observations, the hypothesis that pre-exposure to sand-fly saliva would modify the course of *L. braziliensis* infection was investigated. Surprisingly, mice repeatedly inoculated with *L. intermedia* SGS were not protected but rather showed enhanced disease (53). Moreover, we observed that in an area endemic for *L. braziliensis*, Cutaneous Leishmaniasis (CL) patients displayed higher anti-saliva IgG responses compared with those with a cellular immune response to *Leishmania*. Human monocytes stimulated with *L. intermedia* SGS and exposed to *L. braziliensis* also upregulated TNF-α, IL-6, and IL-8 (54), indicating the ability of *L. intermedia* saliva to alter the inflammatory milieu. Recently, however, we showed that mice immunized with a DNA plasmid coding for Linb11 – a molecule present in *L. intermedia* saliva – displayed reduced parasite load following infection with *L. braziliensis*. If one envisages a combined approach toward vaccine development encompassing parasite and salivary molecules, a careful selection must be made on the latter, as there are molecules that may protect against disease while others may enhance disease (55).

## Identifying the Components of Sand-Fly Saliva: The Age of “Sialomes”

Salivary proteins have been identified in an increasing number of vectors of leishmaniasis in the last two decades (Table 2). So far, transcriptomics and proteomics allowed identification of around 40 salivary proteins such as apyrases, endonucleases, antigen 5-related proteins, D7-like salivary proteins, and yellow proteins – all of which are found in several other organisms (Table 2). Apyrases hydrolyze ATP to ADP and AMP, inhibiting ADP- and collagen-induced platelet aggregation (56–65). Endonucleases cleave DNA, likely decreasing blood-pool viscosity and increasing feeding success (66). In mosquito saliva, D7 salivary proteins are encoded by a multigene family related to the arthropod odorant-binding protein (OBP) superfamily and forms having either one or two OBP domains are found (67). In mosquitoes, D7 proteins act as anti-inflammatory mediators through binding of host biogenic amines, leukotrienes, and AnST-D7L1, present in *Anopheles stephensi*, specifically binds thromboxane A_2_ (TXA_2_) (68). Last, yellow proteins bind to biogenic amines such as serotonin, catecholamine, and histamine, and this binding may dampen the pro-inflammatory response, blocking development of an avert reaction to the bite (69). On the other hand, families such as Lufaxin, an FXa inhibitor (70), Ppsp32-like proteins, and SL1/PpSP15-like proteins were found only in sand flies (71, 72). The functions of D7-like, antigen 5-like, and SL1/PpSP15 families of proteins remain to be determined.

**Table 2:** **Families of most abundant *Phlebotomus* and *Lutzomyia* sand fly proteins**.

Species	Vertebrates		Insect-specific			Sand-fly-specific		
	Apyrase	Endonuclease	Antigen 5	D7	Yellow	SL1/PpSp15	30-kDa Family	Lufaxin
***Phlebotomus***
*P. papatasi* (33)	PpSP36		PpSP29	PpSP28, 30	PpSP46, 44, 42	PpSP15, 14, 12	PpSP32	PpSP34
*P. papatasi* (73) *(Tunis)*	PPtSP36		PPtSP29	PPtSP28, 30	PPtSP42, 44	PPtSP12-15	PPtSP32	PPtSP34
*P. duboscqi* (69) *(Kenya)*	PduK50		PduK68, 107	PduK34–35, 69, 78, 103	PduK04–06, 86	PduK01–03, 40–42, 49, 56–58, 109	PduK45–46, 83	PduK70
*P. duboscqi* (69) *(Mali)*	PduM38–39		PduM48	PduM01, 29, 46, 47, 77	PduM10, 35	PduM02–03, 06–07, 12, 31–32, 49–50, 57–58, 60, 62, 99	PduM72, 33–34, 87	PduM04–05
*P. sergenti* (74)	PsSP40–42		PsSP52	PsSP4, 5, 7	PsSP37, 38	PsSP9-11, 14–15, 54–55	PsSP44	PsSP49
*P. arabicus* (72)	PabSP39–41	PabSP49	PabSP4	PabSP20, 54, 59, 84	PabSP26	PabSP2, 45, 93	PabSP29, 30, 31	PabSP32, 34
*P. tobbi* (74)	PtSP4, 10		PtSP77, 78, 79	PtSP42, 44, 54, 56–58, 60	PtSP18–20, 22, 26	PtSP9, 17–18, 23, 31–32	PtSP27–29	PtSP66
*P. perniciosus* (67)	PpeSP01-B	PpeSP32	PpeSP07	PpeSP04, 04B, 10	PpeSP03, 03B	PpeSP02, 09, 11	PpeSP05	PpeSP06
*P. ariasi* (72)	ParSP01	ParSP10	ParSP05	ParSP07, 12, 16	ParSP04, 04B	ParSP03, 08	ParSP02	ParSP09
*P. argentipes* (67)	PagSP03	PagSP11	PagSP05	PagSP10, 25	PagSP04	PagSP 01, 02, 07, 12–13		PagSP09
***Lutzomyia***
*L. longipalpis* (71)	LJL23	LJL138	LJL34	LJL13	LJM17, LJM11, LJM111	LJM04		LJL143/Lufaxin
*L. intermedia* (70)	Linb-35	Linb-46	Linb-13	Linb-42	Linb-21	Linb-7, 8, 28, 59		Linb-17
*L. ayacuchensis* (69)	LayS 16–21	LayS147	LayS73–80	LayS95–103	LayS22–24, 117, 188	LayS36, 37, 58–72		LayS167, 168
Range of Mw (kDa)	35–36		28.8–31.2	25.3–28.1	41.5–45.2	12.2–17.1	22.5–34.9	32.3–34.5
Highlights	Converts ATP in ADP/AMP				Binds to biogenic amines/protective in mice			Inhibition of factor Xa

Sialomes from *L. intermedia, Lutzomyia ayacuchensis*, and *L. longipalpis* – all found in the New World – express a plethora of 2- to 6-kDa small peptides classified as members of the RGD family of proteins, which may function as inhibitors of platelet aggregation (73, 74). Salivary peptides with an RGD motif are yet to be identified in sand flies from the Old World (73, 74). Linb11, found in *L. intermedia*, seems specific to this species, and immunization with this molecule protected mice from *L. braziliensis* (73). On the other hand, Maxadilan, a powerful vasodilator found in *L. longipalpis* (21), is apparently absent from *L. intermedia* and *L. ayacuchensis*, highlighting differences among the salivary components of different vectors. Additionally, sand flies from the Old World display more abundant transcripts coding for SL1/PpSP15-like proteins, D7-related proteins, and also for yellow proteins compared with New World sand-fly species (71).

Apart from Maxadilan, all other salivary proteins have been identified following a common approach consisting of dissection of salivary glands, isolation of mRNA, cDNA library construction, and sequencing of fewer than 2000 phages (33, 71, 73, 75–78). Bioinformatics have played a major role in identifying salivary transcripts by targeting proteins that bear a signal peptide (SignalP software) (79), thereby differentiating secretory proteins from structural and housekeeping transcripts. This set of techniques advanced our understanding of the most abundant salivary proteins in the absence of a sand-fly genome; however, this methodology has a bias for transcriptomic abundance and hardly detects salivary proteins of large molecular weight. One example is the Hyaluronidases – enzymes that degrade hyaluronic acid, reducing skin viscosity and facilitating blood feeding – commonly found in the venom of bees and wasps. Although hyaluronidase activity has been detected in all sand flies studied so far (78), the current approach mostly failed to detect genes coding for this protein. Hyaluronidase-coding transcripts were found in only 2 of the 13 sand-fly transcriptomes available so far (Table 2). Moreover, endonucleases were not found in *P. duboscqi* and *P. papatasi* transcriptomics but their activity has been detected in salivary glands of both species (Oliveira, unpublished). Advances involving next-generation deep sequencing and sand-fly genome annotation will surely expand the current knowledge of salivary proteins and will allow for identification of a more complete catalog of sand-fly salivary proteins.

## What about Natural Exposure to Sand Flies in Endemic Areas?

Studies in areas endemic for VL in Brazil showed an association between anti-*L. longipalpis* antibodies and the presence of an anti-*Leishmania* cell-mediated immune response in humans (80). Simultaneous development of a humoral response to saliva and a cellular response to *Leishmania* (described by the presence of a positive DTH) (81) suggested that induction of an immune response against *L. longipalpis* SGS could facilitate development of a protective response against leishmaniasis. This notion was strengthened by the demonstration that volunteers experimentally exposed to *L. longipalpis* bites displayed a strong cellular immune response to *L. longipalpis* SGS characterized by IFN-γ production and appearance of a DTH at the bite site (82). Moreover, co-culture of infected macrophages plus autologous lymphocytes in the presence of *L. longipalpis* SGS stimulated a recall response that significantly reduced the parasite load *in vitro*. In a prospective cohort study in a VL-endemic area in Brazil, the incidence of DTH to *Leishmania* antigen was higher among residents with anti-SGS IgG (83), leading to the hypothesis that immunity to *L. longipalpis* sand-fly saliva is a surrogate marker of protection against *L. infantum chagasi* infection. It is unclear whether this protective effect is due to antibodies that neutralize the “exacerbative” properties of sand-fly saliva or due to an anti-saliva cellular immune response that acts rapidly after parasite inoculation, limiting *Leishmania* survival in the human host.

There are, as expected, limitations to using SGS in epidemiologic studies, as sand flies need either to be reared in laboratory colonies or to be actively collected in the field. To this end, the “sialomes” discussed above allowed the identification, cloning, and expression of recombinant salivary proteins from distinct sand-fly species. Employing recombinant salivary proteins, Teixeira et al. showed that foxes and dogs – reservoirs for VL – as well as individuals from an endemic area in Brazil recognize LJM11 and LJM17, present in *L. longipalpis* saliva (84). These results were validated in a sample of 1077 individuals, and detection levels improved significantly when the two proteins were used in combination (85). A prospective study conducted in Tunisia with a cohort of 200 children showed that IgG antibodies (primarily IgG4) against *P. papatasi* SGS were associated with an increased risk of CL caused by *L. major* (86). In a subsequent study, recombinant PpSP32 was described as the immunodominant antigen in the humoral response, acting as a marker of sand-fly exposure (87). In Turkey, patients from a CL-endemic area where *Leishmania tropica* is prevalent displayed significantly higher anti-*Phlebotomus sergenti* IgG levels when compared with healthy individuals from the same area (88). These data suggest that saliva can be used for monitoring exposure of humans to sand flies. Indeed, Clements et al. found a correlation between antibodies to *Phlebotomus argentipes* saliva and vector density (89). Chickens are also useful at monitoring exposure to sand flies, as they develop specific anti-SGS IgY and, therefore, can be employed as sentinel animals (90).

In Mali, where *P. duboscqi* is prevalent, three profiles of cellular immune response were observed: while 23 and 25% of individuals developed a Th1 or Th2-polarized immune response, respectively, 52% displayed a mixed Th1/Th2 response to salivary molecules (91). Analysis of biopsy samples in a subset of anti-saliva DTH-reactive individuals revealed the presence of lymphocytes, macrophages, and IFN-γ production, which were associated with the Th1 response. These results suggest that individuals presenting a Th1-polarized response would be protected against CL, as seen in the experimental models of infection (92), whereas individuals with Th2 immune response remain susceptible to disease. Prospective studies evaluating outcome of infection in these polarized individuals are necessary to further address this hypothesis. On the contrary, in Tunisia, individuals naturally exposed to *P. papatasi* bites displayed antigen-specific IL-10 production by CD8^+^ T cells *in vitro*, while activated CD4^+^ T lymphocytes cultured in the absence of CD8^+^ T cells were able to produce IFN-γ (93). The authors suggested that IL-10 production favors *L. major* proliferation at the moment of transmission by infected sand flies.

## Perspectives

Many factors represent challenges to the study of leishmaniasis, from the shortage of funding to climate change and population displacements. Nevertheless, the almost ubiquitous use of needle infections – bypassing the natural transmission process through an infected sand-fly bite – is a serious limitation. We face the risk of studying a model of disease too disparate from leishmanial disease occurring in the field, thus leading to unsuccessful vaccines and treatments. There is the need to support the establishment of more sand-fly colonies and standardization of techniques for sand-fly infection among the leishmaniasis community. Moreover, more prospective studies in humans from endemic areas are indispensable to better understand the basis of protective human immune responses to *Leishmania* and sand-fly saliva.

## Author Contributions

All authors have discussed and written the manuscript.

## Conflict of Interest Statement

The authors declare that the research was conducted in the absence of any commercial or financial relationships that could be construed as a potential conflict of interest.
